# Silicon-mediated herbivore defence in a pasture grass under reduced and Anthropocene levels of CO_2_

**DOI:** 10.3389/fpls.2023.1268043

**Published:** 2023-11-01

**Authors:** Fikadu N. Biru, Christopher I. Cazzonelli, Rivka Elbaum, Scott N. Johnson

**Affiliations:** ^1^ College of Agriculture and Veterinary Medicine, Jimma University, Jimma, Ethiopia; ^2^ Hawkesbury Institute for the Environment, Western Sydney University, Penrith, NSW, Australia; ^3^ R H Smith Institute of Plant Sciences and Genetics in Agriculture, The Hebrew University of Jerusalem, Rehovot, Israel

**Keywords:** antioxidant enzyme, carbon, carbon dioxide, herbivore, physical defences, plant defences, silicon

## Abstract

The uptake and accumulation of silicon (Si) in grass plants play a crucial role in alleviating both biotic and abiotic stresses. Si supplementation has been reported to increase activity of defence-related antioxidant enzyme, which helps to reduce oxidative stress caused by reactive oxygen species (ROS) following herbivore attack. Atmospheric CO_2_ levels are known to affect Si accumulation in grasses; reduced CO_2_ concentrations increase Si accumulation whereas elevated CO_2_ concentrations often decrease Si accumulation. This can potentially affect antioxidant enzyme activity and subsequently insect herbivory, but this remains untested. We examined the effects of Si supplementation and herbivory by *Helicoverpa armigera* on antioxidant enzyme (catalase, CAT; superoxide dismutase, SOD; and ascorbate peroxidase, APX) activity in tall fescue grass (*Festuca arundinacea*) grown under CO_2_ concentrations of 200, 410, and 640 ppm representing reduced, ambient, and elevated CO_2_ levels, respectively. We also quantified foliar Si, carbon (C), and nitrogen (N) concentrations and determined how changes in enzymes and elemental chemistry affected *H. armigera* relative growth rates and plant consumption. Rising CO_2_ concentrations increased plant mass and foliar C but decreased foliar N and Si. Si supplementation enhanced APX and SOD activity under the ranging CO_2_ regimes. Si accumulation and antioxidant enzyme activity were at their highest level under reduced CO_2_ conditions and their lowest level under future levels of CO_2_. The latter corresponded with increased herbivore growth rates and plant consumption, suggesting that some grasses could become more susceptible to herbivory under projected CO_2_ conditions.

## Introduction

Most grasses are silicon (Si) accumulators, which can account for up to 10% of their dry mass (Epstein, 1994). Si uptake and accumulation are a functional trait with multiple implications for plant biology and ecology (Epstein, 2009). Si is taken up as silicic acid [Si(OH)_4_] via the roots and, after being transported into plant tissues, is deposited within and between plant cells, the cell wall, and silicified structures such as trichomes or other phytoliths (Perry et al., 1984; Kumar et al., 2017). Although the role of Si in protecting plants against multiple biotic (e.g., herbivores and pathogens) and abiotic (e.g., drought and salinity) stresses has been widely reported (Cooke and Leishman, 2011; Debona et al., 2017), an understanding of the exact mechanisms underpinning such protection remains incomplete (Coskun et al., 2019). However, the consensus is that Si supplementation enhances plant physical defences and integrates with the regulation of secondary metabolite defences (Reynolds et al., 2016; Alhousari and Greger, 2018; Hall et al., 2019; Waterman et al., 2020).

In terms of physical defences, it is well established that Si confers plant resistance and reduces plant damage caused by both vertebrate and invertebrate herbivores (Massey and Hartley, 2009; Hartley et al., 2015; Alhousari and Greger, 2018). Si deposition within and around plant cells makes plant tissues tougher and abrasive, causing wear on herbivore mouthparts, damages digestive organs, inhibits movement, and reduces the feeding efficiency of insect herbivores (Massey et al., 2006; Reynolds et al., 2009). Moreover, Si is known to alter grass physical defences such as macrohairs, silica cells, and prickle cells, which are linked to reduced feeding by insect herbivores (Hartley et al., 2015; Hall et al., 2020; Biru et al., 2021). Si uptake and accumulation have also been shown to be induced following herbivory (Massey et al., 2007; Islam et al., 2020; Biru et al., 2022).

In addition to direct physical defences, Si potentially protects plants against herbivores by influencing production of plant biochemical defences (Reynolds et al., 2016; Yang et al., 2017), although there is much uncertainty about this since Si has limited chemical reactivity within the plant. Herbivore attack is associated with the induction of oxidative stress in plants, resulting from overproduction of reactive oxygen species (ROS) (Bi and Felton, 1995; Kerchev et al., 2012). For instance, insect herbivore attacks can induce various ROS such as hydrogen peroxide (H_2_O_2_), superoxide (O_2_•−), singlet oxygen (^1^O_2_), or hydroxyl radicals (•OH) in cells (Sharma et al., 2012; Das and Roychoudhury, 2014). While ROS have signalling roles under physiological setup (Hasanuzzaman et al., 2020), (biotic) stress-induced ROS overproduction damages cell structures and functionality (Tripathy and Oelmüller, 2012; Das and Roychoudhury, 2014; Fichman and Mittler, 2020). In order to reduce excessive ROS content caused by the imbalance between free radical formation and the capability of cells to detoxify them (Pizzino et al., 2017), plants have developed efficient antioxidant enzymatic machinery to scavenge ROS (Tripathy and Oelmüller, 2012). The antioxidant defence system in the plant cell includes both enzyme constituents such as superoxide dismutase (SOD), catalase (CAT), ascorbate peroxidase (APX), glutathione reductase (GR), and non-enzyme constituents like cysteine (Cys), reduced glutathione (GSH), and ascorbic acid (AsA) (Farooq et al., 2013; Kim et al., 2017). Plants possess either constitutive or induced antioxidants (Sudhakar et al., 2001) and the increased activities of these enzymes in plant cells appear to better control oxidative stress (Das and Roychoudhury, 2014; Huang et al., 2019).

Exogenous Si application has been linked to enhanced activity of antioxidant enzyme defences such as CAT, APX, and SOD (Kim et al., 2016; Hasanuzzaman et al., 2018; Ahanger et al., 2020). However, the mode of action by which Si regulates antioxidant capacities is poorly understood. Previous work has shown that Si enhances SOD, CAT, APX, and peroxidase activities in rice (*Oryza sativa*) (Han et al., 2016), wheat (*Triticum aestivum* L.) (Gong et al., 2005), and maize (*Zea mays* L.) (Moussa, 2006). Si-mediated regulation of antioxidant defences, therefore, reduces the harmful effects of herbivore-induced oxidative stress (Ahanger et al., 2020; Acevedo et al., 2021).

Atmospheric concentrations of carbon dioxide (CO_2_) have emerged as an important environmental driver of Si accumulation (Johnson and Hartley, 2018). In general, several studies report that elevated CO_2_ concentrations (eCO_2_) decrease Si accumulation (Ryalls et al., 2017; Johnson and Hartley, 2018; Johnson et al., 2023), but see Frew et al. (2017) and Fulweiler et al. (2014). In contrast, reduced levels of atmospheric CO_2_ can lead to increased Si accumulation (Biru et al., 2020; Biru et al., 2021). These effects are likely due to carbon (C) being either more available under eCO_2_ (Johnson and Hartley, 2018; Johnson et al., 2022) or less available under reduced CO_2_ conditions (Biru et al., 2020). Si accumulation is often negatively correlated with C potentially due to stoichiometric dilution or Si substitution for C-based structural or defensive compounds (Raven, 1983; Hodson et al., 2005).

Given CO_2_ is such an important driver of Si accumulation, which has been shown to influence enzymatic responses (e.g., Kim et al., 2016; Ahanger et al., 2020; Acevedo et al., 2021), CO_2_ may also affect production of plant biochemical defences (i.e., antioxidant enzyme), potentially via enhanced plant susceptibility to herbivore-induced oxidative stress. To our knowledge, no studies have yet investigated the effects of variable rates of Si accumulation on antioxidant enzyme activity and regulation of herbivore-induced oxidative stress under different CO_2_ concentrations. Using tall fescue (*Festuca arundinacea*) and the generalist insect herbivore, cotton bollworm [*Helicoverpa armigera* (Hübner)], we investigated the effect of Si treatments on antioxidant enzyme activities and foliar chemistry of plants grown under three CO_2_ concentrations (200, 410, and 640 ppm) and the consequences for insect herbivory. The objective of this study was to determine how Si treatments (+Si or −Si) under different CO_2_ concentrations affect antioxidant enzyme activity and foliar chemistry (e.g., C, N) in tall fescue and the consequences for insect herbivore growth rate and feeding efficiency. We hypothesised that (1) +Si and reduced CO_2_ decrease shoot C concentrations but increase shoot N concentration, i.e., decreasing shoot C-to-N ratio, and (2) +Si together with reduced CO_2_ treatments increases antioxidant enzyme activity, whereas −Si together with elevated CO_2_ would decrease antioxidant enzyme activity.

## Materials and methods

### Plant material and growth conditions

Tall fescue (*Festuca arundinacea*) is a common pasture grass and a high Si accumulator (Hodson et al., 2005). Seeds of tall fescue (accession T 9627), obtained from Margot Forde Germplasm Centre (Palmerston North, New Zealand), were sterilised in a solution of 0.9% sodium hypochlorite and 0.1% Triton X-100 for 30 min, followed by washing several times with water before being inserted into perlite irrigated with water. Seeds were stratified at 4°C for 3 days, and plants were grown in the glasshouse for 2 weeks using a rectangular plastic tray to achieve uniform seedling growth. Two weeks after germination, individual plants were transferred to hydroponics cups. The hydroponics setup consisted of two nested disposable plastic cups as per Hall et al. (2019). Each cup was filled with approximately 350 mL of full-strength standard hydroponic solutions following the protocol of Jung et al. (2015). Seedlings were grown in three plant growth chambers (TPG-1260TH, Thermoline Scientific, NSW, Australia), maintained at a reduced CO_2_ of 200 ppm, an ambient CO_2_ of 410 ppm, and an elevated CO_2_ of 640 ppm CO_2_ concentrations; the latter CO_2_ concentration predicted for 2100 under the RCP6.0 scenario outlined by the IPCC (2014). Chambers were illuminated with five 400-W Sunmaster Dual Spectrum High-Pressure Sodium globes at 350 µmol m^−2^ s^−1^ at the plant canopy level. Daytime air temperature was regulated at 26°C and fell to 18°C at night on a 15L:9D photoperiod cycle. Humidity was controlled at 50% ( ± 6%). Carbon dioxide within the chambers was monitored by a Li-Cor LI-820 CO_2_ gas analyser, with CO_2_ (food grade, Air Liquide, NSW, Australia) injected from pressurised cylinders through solenoid valves. For reduced CO_2_ treatment in the chamber, the computer controller constantly monitors CO_2_ and powers fans to direct chamber air through the scrubbers filled with Sodasorb^®^ (W.R. Grace & Co, Chicago, USA).

### Experimental design

**Figure 1:**
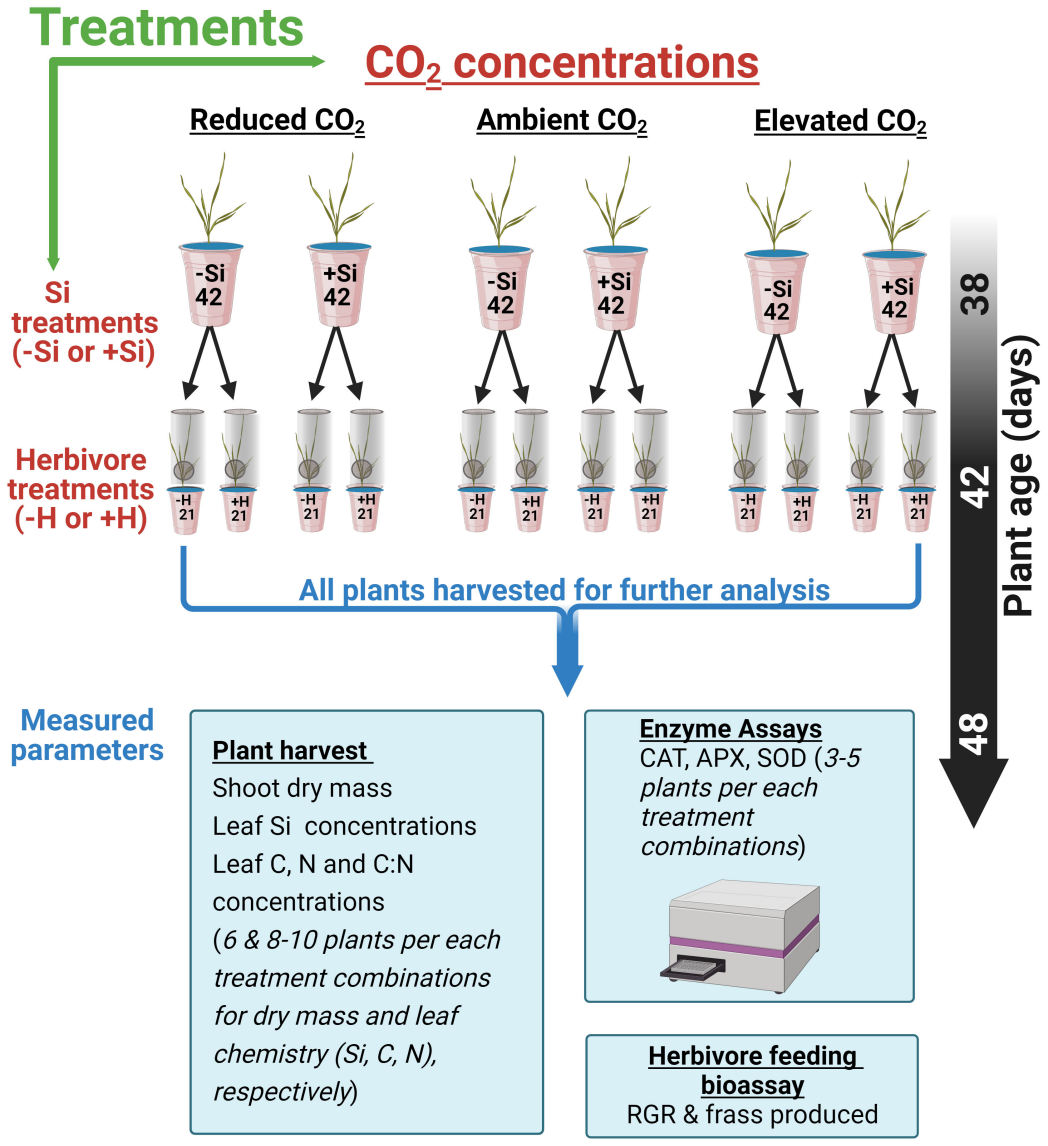
Schematic showing 252 (42*6) fescue grass grown under three CO_2_ concentrations and Si treatments (−Si or +Si). Six weeks after transplant, plants were either inoculated with herbivore, (+H) or kept in control (−H). The specific arrangement of the cup within each chamber was randomised and swiped within the chambers every 2 weeks.

The experimental design consisted of 252 hydroponically grown tall fescue plants. The experiment comprised a factorial combination of CO_2_ concentrations (200, 410, or 640 ppm), Si (+Si or −Si), and herbivore (herbivory, +H; no herbivory, −H) treatments (see **Figure 1**). Si treatments (+Si) used liquid potassium silicate (K_2_SiO_3_) (Agsil32, PQ Australia, SA, Australia) at a concentration equivalent to 2 mM SiO_2_. Chemically, silicic acid polymerises to form silica gel when the concentration of silicic acid exceeds 2 mM (Ma and Yamaji, 2006). Potassium chloride was added to the control (−Si) cups to balance additional K^+^ ions in +Si treatments. The pH of both +Si and −Si solutions was adjusted to 5.6 using hydrochloric acid to reduce silicate polymerisation (Ma and Yamaji, 2006). Solutions were replaced weekly for the first 2 weeks and then three times a week afterwards. Cups were rotated and chambers were swapped weekly to minimise chamber effects and pseudo-replication as previously described by Johnson et al. (2018). Plants were grown hydroponically for a further 6 weeks (42 days, **Figure 1**) before insect inoculation.

### Herbivore performance

To assess the impacts of different CO_2_ concentrations and Si supplementation on the growth of *H. armigera* larvae, a feeding performance assay was conducted. *Helicoverpa armigera* third instar larvae supplied by CSIRO Agriculture & Food, Narrabri, Australia, reared on an artificial diet (Teakle and Jensen, 1985), were used for feeding assays. Initially, larvae were starved for 24 h and weighed, and a single larva was either applied for each plant shoot [herbivory (+H)]) or kept control [no herbivory (−H)] (see **Figure 1** for details). Each plant was then caged with transparent Perspex sheaths and herbivores were allowed to feed on shoot parts of the plants for 6 days, after which they were removed and starved for a further 24 h to allow the frass to pass, before being reweighed. All frass were collected, dried, and weighed. Frass production was used as a surrogate for plant consumption. RGR was calculated according to Massey and Hartley (2009). RGR estimates the change in larval fresh mass relative to initial mass and was calculated as mass gained (mg)/initial mass (mg)/time (days).

### Antioxidant enzyme activity assays

Immediately after herbivore removal, plants from all treatment groups were harvested into liquid nitrogen and stored at −80°C until analysis. For the measurement of enzymatic activities, ca. 0.05 g of leaf tissue was ground in liquid N and homogenised in 3 mL of ice-cold 100 mM K-phosphate buffer (pH 6.8) containing 0.1 mM EDTA. The homogenate was centrifuged at 16,000 *g* for 15 min and the supernatant was used as the source of crude extracts. The supernatant was utilised to measure the activity of antioxidant enzyme such as CAT, APX, and SOD.

CAT activity was measured spectrophotometrically following the method of Fimognari et al. (2020) and Maksimović and Živanović (2012). Reaction mixture consists of 50 mM potassium phosphate buffer, pH 7.0, 20 mM H_2_O_2_, and crude extract. Absorbance at 240 nm was recorded for 130s using CLARIOstar^®^ plate reader (BMG Labtech, Ortenberg, Germany) in 96-well plates (96-well Flat Bottom Plate, Greiner Bio-one, Australia). For control reactions, H_2_O_2_ was omitted. Enzyme activity was calculated using the molar extinction coefficient 36 × 10^3^ mM^−1^ m^−1^ and expressed as µmol H_2_O_2_ oxidised g^−1^ FW min^−1^.

APX activity was assayed according to Hartmann and Asch (2019). The reaction mixture consists of 50 mM potassium phosphate buffer, pH 7.0, 0.2 mM ascorbate, 0.2 mM H_2_O_2_ and crude extract. Absorbance at 290 nm was recorded for 130 s using CLARIOstar^®^ in 96-well plates. APX activity was calculated according to Hartmann and Asch (2019); one unit of APX is defined as the amount of enzyme required to oxidise 1 μmol of ascorbic acid per minute.

SOD activity was estimated following the inhibition of photochemical reduction of nitroblue tetrazolium (NBT) by the enzyme according to Hartmann and Asch (2019). The reaction mixture contained 0.05 M sodium carbonate, 13.3 mM methionine, 1.3 μM riboflavin, 21 μM NBT, and plant extract (Hartmann and Asch, 2019). The reaction took place in a chamber under illumination of a 30-W fluorescent lamp at 25°C. The reaction was started by turning the fluorescent lamp on and stopped 5 min later by turning it off. The blue formazan produced by NBT photoreduction was measured as increase in absorbance at 560 nm. The blank solution had the same complete reaction mixture but was kept in the dark. One SOD unit was defined as the amount of enzyme required to inhibit 50% of the NBT photoreduction in comparison with wells lacking the plant extract and expressed as units of enzyme activity g^−1^ FW min^−1^ (Cavalcanti et al., 2004).

### Analyses of foliar chemistry

Harvested sample leaves were oven-dried for 3 days at 60°C and ball milled for further analysis (see sample size from **Figure 1**). For foliar Si analysis, roughly 80 mg of ground leaf material was analysed using x-ray fluorescence spectrometry (Epsilon 3^x^, PANalytical, EA Almelo, The Netherlands) as per Reidinger et al. (2012). Si measurements were calibrated against a certified plant reference material of known Si concentrations (Hiltpold et al., 2017). For foliar C and N concentration, approximately 7 mg of ground leaf material was analysed using Elementar Vario EL Cube, CHNOS elemental analyser (Elementar Analysensysteme GmbH, Hanau, Germany), at a combustion temperature of 950°C.

### Statistical analysis

All data were analysed using SPSS (version 27) statistical software. Before analysis, all data were checked for assumptions of normality for residuals according to inspection of quantile–quantile plots. Plant dry mass was analysed on square-root transformed data whereas CAT, C:N ratio, and RGR were analysed on log10 transformed data, as they did not meet the assumptions of normality. Foliar Si was analysed using two-way analysis of variance (ANOVA) type = II, comparing CO_2_, and herbivory (larval fed vs. undamaged controls) as treatments and their interaction. For foliar Si analyses, control (−Si) plants were omitted since −Si plants had Si concentrations lower than the machine detection limits (Reidinger et al., 2012). Plant dry mass, antioxidant enzyme activities (CAT, APX, and SOD), foliar C, N, and C-to-N ratio concentrations were all analysed using three-way ANOVA type = II, comparing CO_2_, Si (Si-supplemented vs. non-Si-supplemented plants) and herbivory as treatments and their interaction. Additionally, we tested the independent effects of CO_2_ on antioxidant enzyme using a one-way ANCOVA, with CO_2_ levels as a fixed factor and foliar Si concentration fitted as a covariate. For herbivore RGR and frass produced, three insects escaped, so data were analysed using type = III ANOVAs due to the unbalanced design. Bonferroni *post hoc* test (Aslam and Albassam, 2020) was applied for pairwise multiple comparisons when interaction terms were statistically significant. Potential relationships between foliar Si and herbivore RGR, frass produced, CAT, APX, and SOD enzymes activity were investigated using Spearman’s rank correlation test.

## Results

### Plant dry mass and foliar chemistry

**Figure 2:**
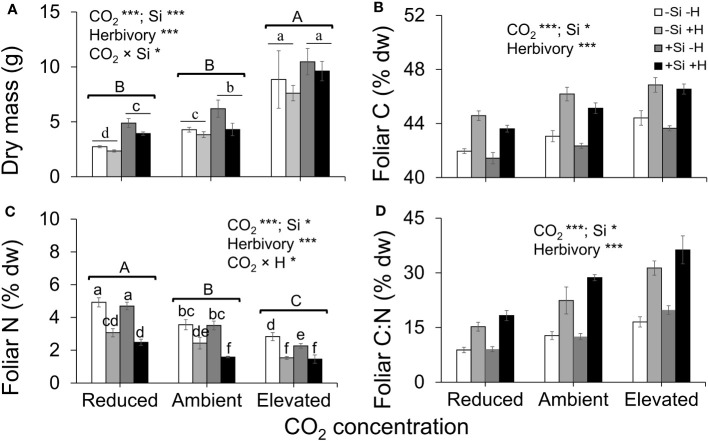
Effects of Si supply (+Si or –Si) and herbivory treatments (+H or –H) on **(A)** plant dry mass, **(B)** foliar C, **(C)** foliar N, and **(D)** foliar C:N of tall fescue grass grown under reduced, ambient, and elevated CO_2_ concentration. Means ± SE shown. *N* = 6–8. Uppercase letters represent differences between CO_2_ concentrations whereas lowercase letters indicate differences between Si treatments (Panel 1A) and herbivore treatments (Panel 1C). Statistically significant effects are indicated *p < 0.05, and ***p < 0.001.

**Figure 3:**
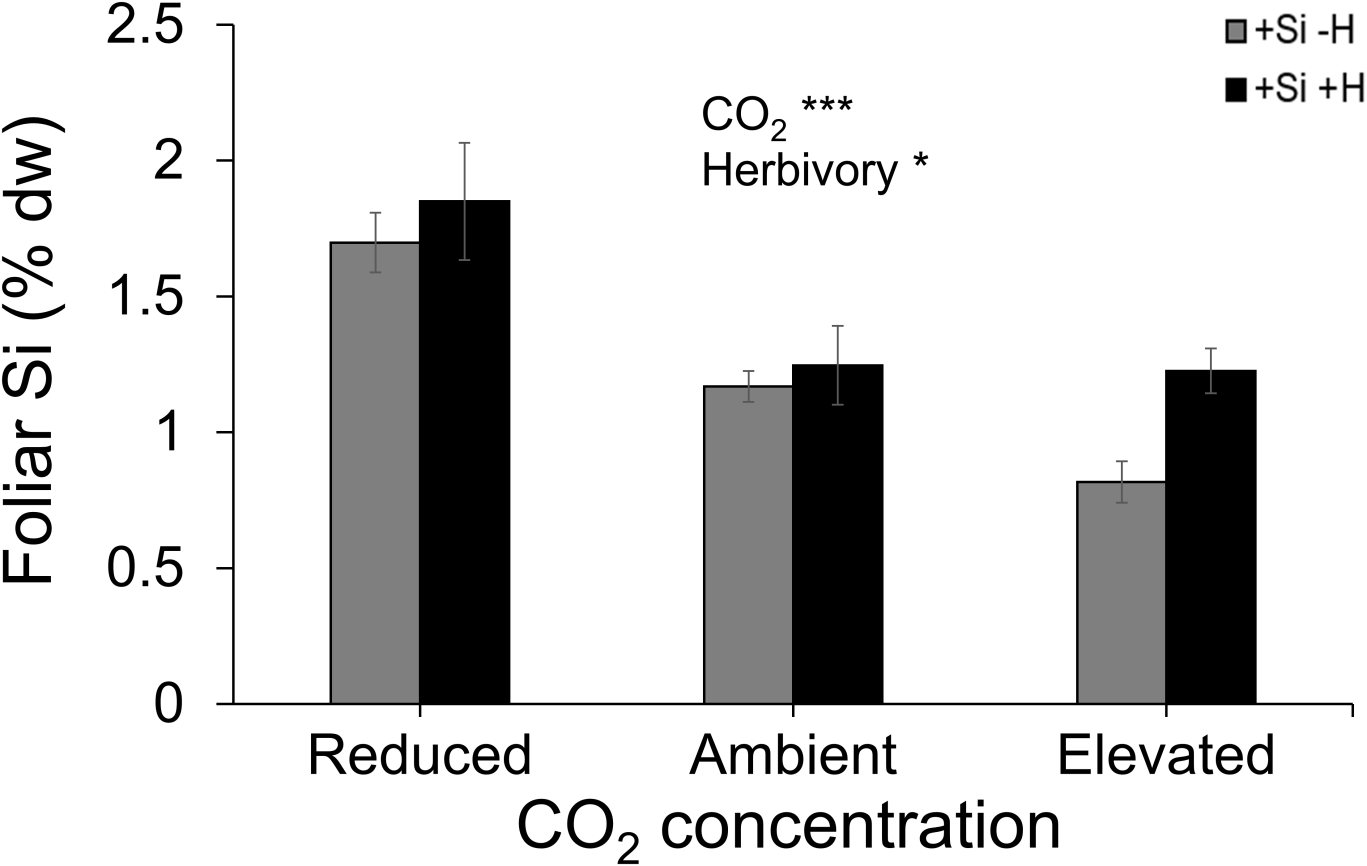
Effects of Si supply and herbivory (+H or –H) on foliar Si concentration of tall fescue grass grown under reduced, ambient, and elevated CO_2_ concentration. Means ± SE shown. *N* = 10. Statistically significant effects are indicated *p < 0.05, and ***p < 0.001.

Averaged across CO_2_ treatments, Si supply increased plant dry mass by 160% relative to those grown without Si supply, whereas elevated CO_2_ increased plant dry mass by twofold and threefold compared to reduced CO_2_ and ambient CO_2_, respectively (**Figure 2A**; **Table 1**). Furthermore, herbivory decreased plant dry mass in Si-free (control) plants by 1.5-, 2-, and 2-fold under reduced, ambient, and elevated CO_2_, respectively, compared to Si-supplemented plants (**Figure 2A**). Si supplementation decreased foliar C concentrations under all CO_2_ regimes. This effect was reversed when herbivores were present and foliar C concentrations increased to levels observed in Si-free plants (**Figure 2B**; **Table 1**). Si supply decreased foliar C by 149%, 177%, and 107% under reduced, ambient, and elevated CO_2_, respectively, regardless of herbivore treatments (**Figure 2B**). Reduced CO_2_ significantly decreased foliar C (**Figure 2B**; **Table 1**). Si supply decreased foliar N under all CO_2_ regimes. However, there was also an effect of CO_2_ whereby reduced CO_2_ increased foliar N by twofold and threefold compared to ambient and elevated CO_2_, respectively, irrespective of herbivore treatments (**Figure 2C**; **Table 1**). In addition to variations in C and N concentrations, there was also an effect on their ratio. Si supply increased foliar C:N ratio especially when plants were damaged by herbivores or in herbivore-free plants only under elevated CO_2_ (**Figure 2D**; **Table 1**). While herbivory increased foliar C:N ratio regardless of CO_2_ levels, reduced CO_2_ decreased foliar C:N ratio relative to elevated CO_2_ (**Figure 2D**; **Table 1**). Foliar Si accumulation [% dry weight (DW)] was significantly higher under reduced CO_2_ relative to ambient and elevated CO_2_ (**Figure 3**; **Table 1**).

**Table 1:** Results of ANOVA for plant biomass, foliar chemistry, and antioxidant enzyme as affected by CO_2_ levels, Si treatment, and herbivore presence and their interactive effects.

Response variables	Figures	Factors						
		CO_2_	Si	Herbivory	CO_2_ × Si	CO_2_ × Herbivory	Si × Herbivory	CO_2_ × Si × Herbivory
Biomass and foliar chemistry								
Biomass^z^	**1A**	F_2,60 =_ 153.1 *p* < **0.001**	*F*_1,60 =_ 44.61 *p* < **0.001**	*F*_1,60 =_ 14.23 *p* < **0.001**	*F*_2,60 =_ 5.761 *p* = **0.005**	*F*_2,54 =_ 0.545 *p* = 0.582	*F*_1,60 =_ 0.489 *p* = 0.487	*F*_2,60 =_ 1.043 *p* = 0.359
C^x^	**1B**	*F*_2,84 =_ 41.34 *p* < **0.001**	*F*_1,84 =_ 10.54 *p* = **0.002**	*F*_1,84 =_ 145.5 *p* < **0.001**	*F*_2,84 =_ 0.211 *p* = 0.801	*F*_2,84 =_ 0.515 *p* = 0.599	*F*_1,84 =_ 0.062 *p* = 0.804	*F*_2,84 =_ 0.409 *p* = 0.666
N^x^	**1C**	*F*_2,84 =_ 54.73 *p* < **0.001**	*F*_1,84 =_ 7.973 *p* = **0.006**	*F*_1,84 =_ 123.1 *p* < **0.001**	*F*_2,84 =_ 0.072 *p* = 0.930	*F*_2,84 =_ 4.151 *p* = **0.019**	*F*_1,84 =_ 0.645 *p* = 0.424	*F*_2,84 =_ 1.856 *p* = 0.163
C:N^z^	**1D**	*F*_2,84 =_ 62.33 *p* < **0.001**	*F*_1,84 =_ 7.512 *p* = **0.007**	*F*_1,84 =_ 154.7 *p* < **0.001**	*F*_2,84 =_ 0.102 *p* = 0.903	*F*_2,84 =_ 0.146 *p* = 0.864	*F*_1,84 =_ 1.818 *p* = 0.181	*F*_2,84 =_ 1.462 *p* = 0.237
Si^a^	**2**	*F*_2,54 =_ 19.31 *p* < **0.001**	────	*F*_1,54 =_ 4.267 *p* = **0.044**	────	*F*_2,54 =_ 0.949 *p =* 0.393	────	────
Antioxidant enzyme activity								
CAT^y^	**3A**	*F*_2,45 =_ 19.19 *p* < **0.001**	*F*_1,45 =_ 5.672 *p =* **0.022**	*F*_1,45 =_ 4.290 *p* = **0.044**	*F*_2,45 =_ 3.843 *p =* **0.029**	*F*_2,45 =_ 0.649 *p* = 0.528	*F*_1,45 =_ 0.000 *p* = 0.989	*F*_2,45 =_ 0.007 *p* = 0. 993
APX^x^	**3B**	*F*_2,34 =_ 13.69 *p* < **0.001**	*F*_1,34 =_ 33.36 *p* < **0.001**	*F*_1,34 =_ 20.57 *p* < **0.001**	*F*_2,34 =_ 1.218 *p* = 0. 308	*F*_2,34 =_ 0.199 *p* = 0.820	*F*_1,34 =_ 0.054 *p* = 0.818	*F*_2,34 =_ 0.066 *p* = 0.936
SOD^x^	**3C**	*F*_2,45 =_ 7.489 *p* = **0.002**	*F*_1,45 =_ 8.781 *p* = **0.005**	*F*_1,45 =_ 2.047 *p =* 0.159	*F*_2,45 =_ 0.061 *p* = 0.941	*F*_2,45 =_ 0.018 *p* = 0.983	*F*_1,45 =_ 0.109 *p* = 0.743	*F*_2,45 =_ 0.094 *p* = 0.910
Herbivore performance								
RGR^a^	**5A**	*F*_2,39 =_ 6.067 *p* = **0.005**	*F*_1,39 =_ 10.03 *p* = **0.003**	────	*F*_2,39 =_ 0.224 *p* = 0.801	────	────	────
Frass^a^	**5B**	*F*_2,39 =_ 2.510 *p* = 0.094	*F*_1,39 =_ 4.825 *p* = **0.034**	────	*F*_2,39 =_ 0.635 *p* = 0.536	────	────	────

a Analysed using a two-way ANOVA.

x Analysed using a three-way ANOVA.

y Analysed using a three-way ANOVA on square-root transformed data.

z Analysed using a three-way ANOVA on log10 transformed data. Statistically significant factors at *p*-values <0.05 are indicated in bold.

### Antioxidant enzyme activity was enhanced by Si uptake and reduced CO_2_

**Figure 4:**
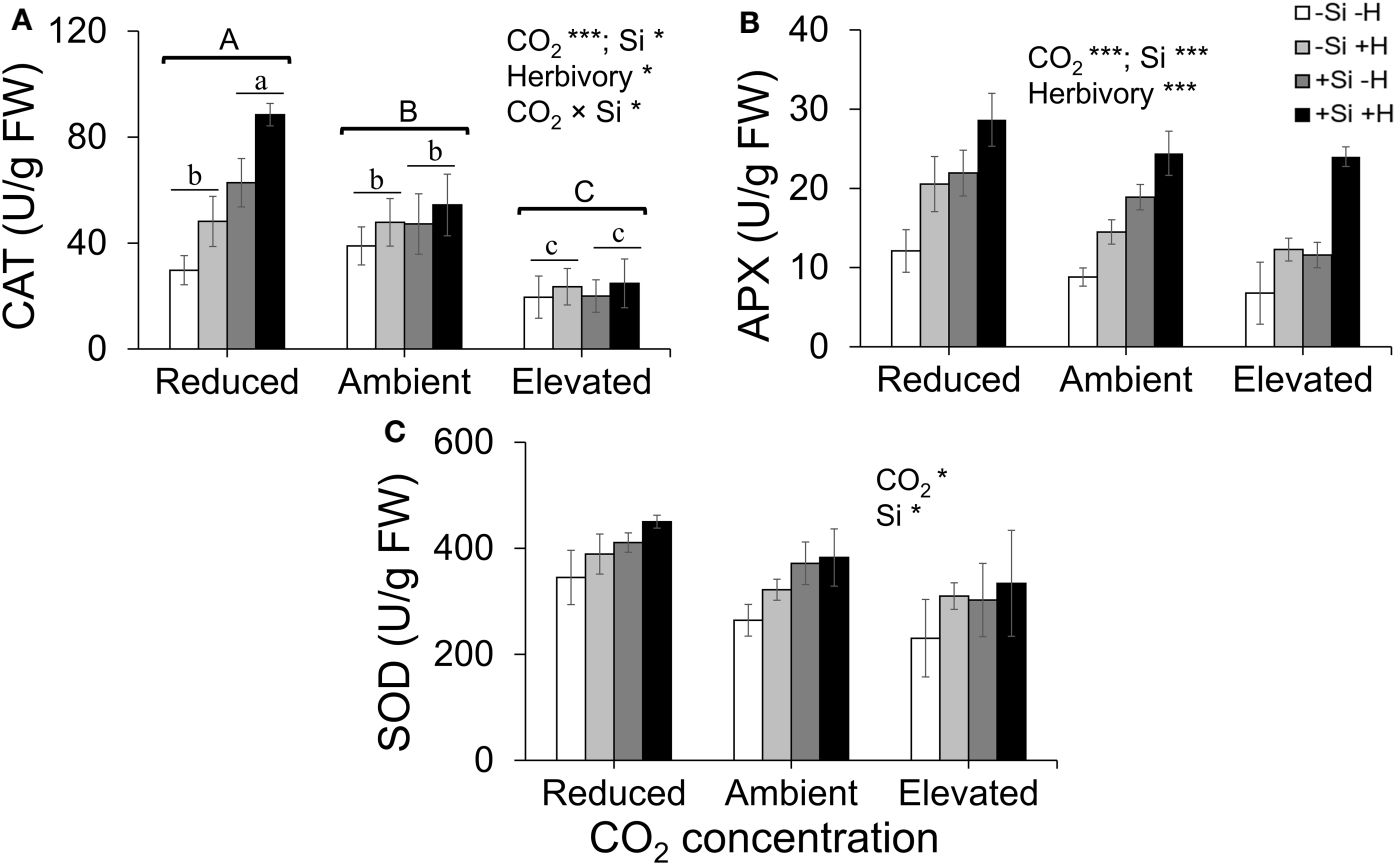
Effects of Si supply (+Si or –Si) and herbivory (+H or –H) on **(A)** CAT, **(B)** APX, and **(C)** SOD enzyme activity of tall fescue grass grown under reduced, ambient, and elevated CO_2_ concentration. Means ± SE shown. *N* = 3–5. Uppercase letters represent differences between CO_2_ concentrations whereas lowercase letters indicate differences between Si treatments (Panel 1A). Statistically significant effects are indicated *p < 0.05, and ***p < 0.001

**Figure 5:**
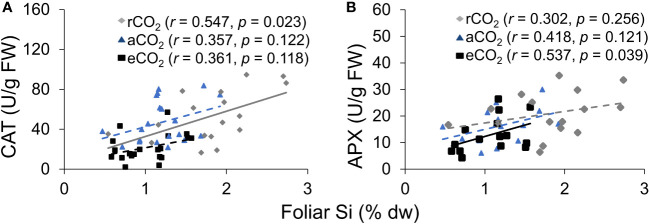
The relationship between **(A)** CAT and foliar Si, and **(B)** APX and foliar Si. The solid line represents linear regression through all data points and dashed lines indicate that no significant relationship was observed.

CAT activity increased in response to Si supply and herbivore damage overall, although this was only apparent under reduced CO_2_ concentrations (**Table 1**; **Figure 4A**). The significant interaction between Si and CO_2_ reflects that Si impacts were only apparent under reduced CO_2_ concentrations (**Table 1**). In contrast, Si supply increased APX enzymes activity under all CO_2_ regimes and SOD enzyme activity under reduced and ambient CO_2_ (**Table 1**; **Figures 4B, C**). Herbivory caused higher APX activity specifically in Si-supplemented plants (**Table 1**; **Figure 4B**); however, it had no significant effect on SOD enzyme activity (**Table 1**; **Figure 4C**). Overall, antioxidant enzyme activity (CAT, APX, and SOD) declined with increasing CO_2_ concentrations (**Table 1**; **Figures 3A–C**). Including foliar Si as a covariate in ANCOVA indicated that the changes in antioxidant enzyme activity were linked to CO_2_ levels, which fully explained the observed changes in CAT (*F*_1,53 =_ 11.67, *p* = 0.001) and APX (*F*_1,42 =_ 5.79, *p* = 0.021) but not in SOD (*F*_1,53 =_ 1.130, *p* = 0.258). There was a positive correlation between foliar Si concentrations and concentration of CAT under reduced CO_2_, and concentration of APX under elevated CO_2_ (**Figures 5A, B**). Interestingly, frass produced was negatively correlated with SOD (*r* = −0.310, *p* = 0.038), but had marginally non-significant negative correlation with APX (*r* = −0.286, *p* = 0.057). However, there was no such relationship observed between CAT and frass produced (*r* = −0.191, *p* = 0.208) (data not shown).

### Si supply and low CO_2_ environment suppressed herbivore RGR and feeding efficiency

**Figure 6:**
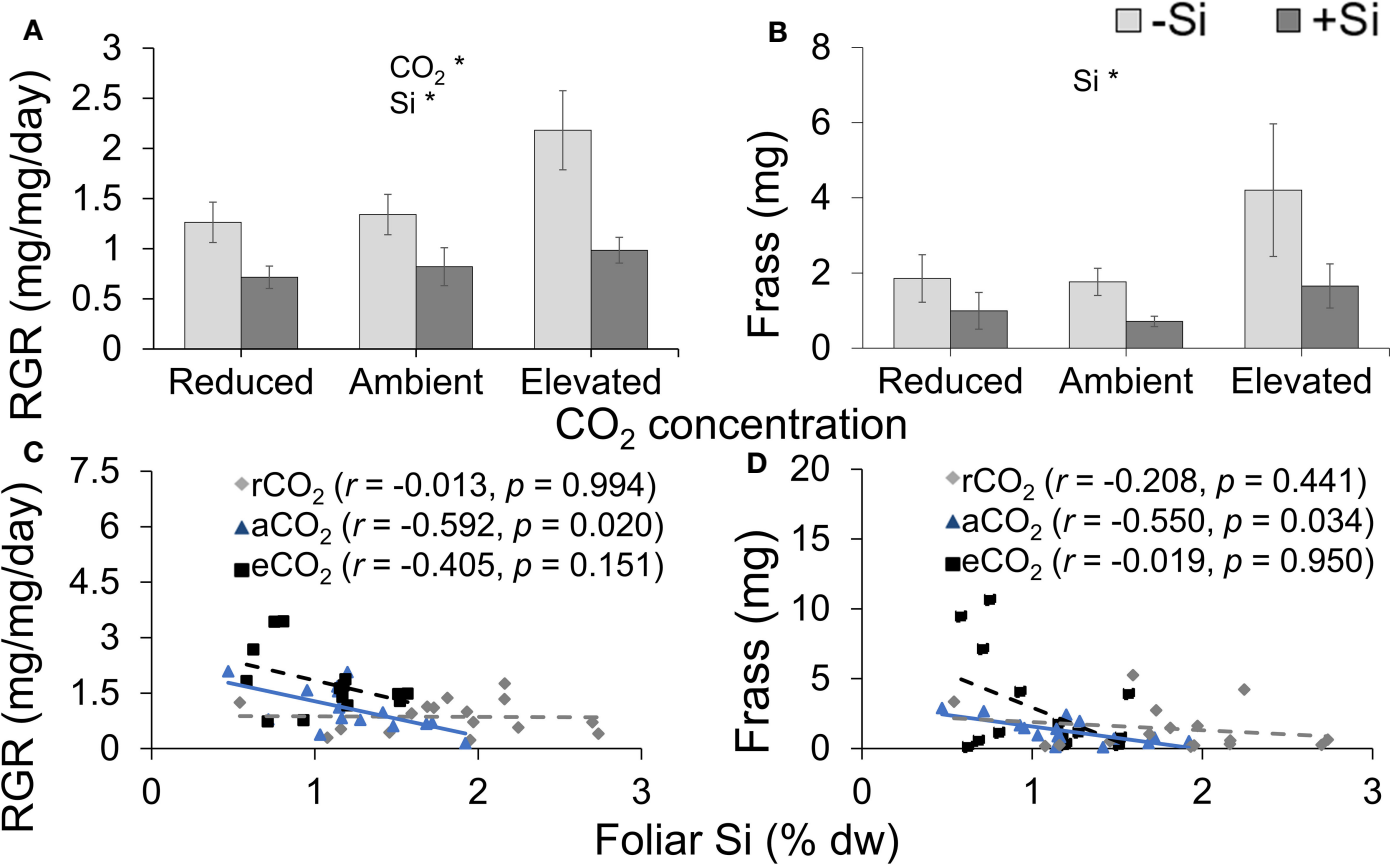
Effects of Si supply (+Si or –Si) and CO_2_ level on **(A)** relative growth rate (RGR) of *Helicoverpa armigera* fed on tall fescue grass, **(B)** frass produced, and the relationship between foliar Si concentrations and **(C)** RGR and **(D)** frass produced. Means ± SE shown. *N* = 7–8. For **(C)** and **(D)**, the solid line represents linear regression through all data points and dashed lines indicate that no significant relationship was observed. Statistically significant effects are indicated *p < 0.05.

Si supplementation decreased RGR under all CO_2_ regimes; RGR was significantly lower under reduced CO_2_ compared to elevated CO_2_ (**Table 1**; **Figure 6A**). Si supply decreased the amount of frass produced by caterpillars (indicative of feeding efficiency) under all CO_2_ levels; CO_2_ had no significant effect on frass production, although there was a large increase in production in Si-free plants grown under elevated CO_2_ (**Table 1**; **Figure 6B**). While herbivore RGR and frass produced were negatively correlated with foliar Si under ambient CO_2_, there was no such relationship observed under the other two CO_2_ regimes (**Figures 6C, D**). Here, the correlation between rate of herbivore feeding on foliar tissues of tall fescue grown under different CO_2_ concentrations potentially indicates a new perspective towards mitigating challenges of CO_2_ enriched environment on plant defences.

## Discussion

**Figure 7:**
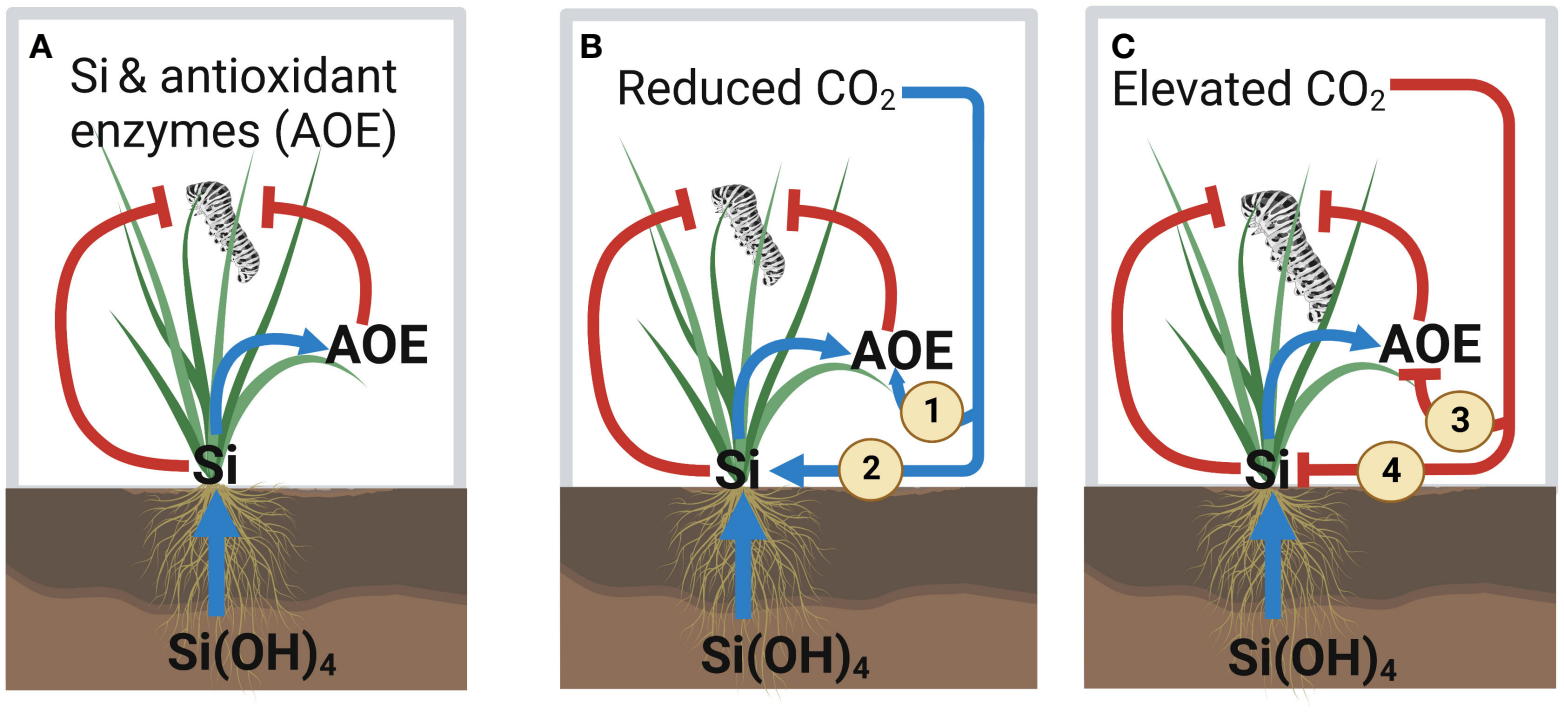
Summary of the effect of silicon (Si) and variable atmospheric CO_2_ concentrations on the Si and antioxidant enzyme defences against herbivory. **(A)** Current knowledge of the interaction effect between Si and antioxidant enzyme (AOE) activity on insect herbivory. Our key results from this study are indicated in panels **(B, C)**. **(B)** Reduced CO_2_ enhances AOE defences by (1) directly enhancing AOE activity and (2) indirectly increasing Si uptake, which leads to reduced herbivore performance. **(C)** Elevated CO_2_ reduces AOE defences by (3) directly reducing AOE activity and (4) indirectly decreasing Si uptake, which leads to increased herbivore performance. Positive and negative effects for both plants and insects are indicated by blue arrows and red lines, respectively.

We demonstrated that reduced levels of atmospheric CO_2_ caused plants to accumulate more Si and produce higher levels of antioxidant enzyme relative to future levels of atmospheric CO_2_. These increased levels of Si and antioxidant enzyme concentrations under reduced levels of CO_2_ were associated with reduced insect herbivore performance. In contrast, herbivore performance and plant consumption (frass production as proxy) were highest under elevated atmospheric CO_2_ conditions, which typically had the lowest levels of Si and antioxidant enzyme. To our knowledge, this is the first study to address the relationship between Si defences and antioxidant enzyme production in the context of variable atmospheric CO_2_ conditions, which we summarise in **Figure 7**.

### Direct and Si-mediated impacts of CO_2_ on the activity of antioxidant enzyme

The effects of CO_2_ in this study on Si concentrations are broadly similar to the few studies exploring this, whereby elevated CO_2_ leads to decreased Si accumulation (Ryalls et al., 2017; Johnson and Hartley, 2018; Biru et al., 2022; Johnson et al., 2022; Johnson et al., 2023), whereas reduced CO_2_ leads to increased Si accumulation. To the best of our knowledge, the current study is only the third study to investigate the latter (Biru et al., 2020; Biru et al., 2021). Si has been hypothesised to act as a structural substitute for C at a lower metabolic cost, particularly when CO_2_ concentrations were lower in the Miocene (Craine, 2009).

Exogenous application of Si has been shown to increase antioxidant enzyme including CAT, APX, and SOD (Kim et al., 2016; Hasanuzzaman et al., 2018; Ahanger et al., 2020). It seems likely that changes in Si in response to CO_2_ growing conditions influenced activity of antioxidant enzyme in the current study. Additionally, the ANCOVA results indicated that CO_2_ affects antioxidant enzyme activity (e.g., CAT and APX), which were mostly explained by the direct impacts of CO_2_ levels on antioxidant enzyme. However, the observed positive correlation of leaf Si with CAT and APX under reduced CO_2_ and elevated CO_2_, respectively, suggests that CAT defence response may be linked to higher levels of Si under reduced CO_2_, whereas APX defence response may be associated with increased induction of defence responses following herbivore attack under elevated CO_2_. Overall, our results demonstrated significant augmentation of antioxidant enzyme responses via increased Si uptake under reduced CO_2_ as well as by the direct effect of CO_2_ levels (see **Figure 7**). In contrast, previous studies have reported that elevated CO_2_ increases activity of antioxidant enzyme in different plants (Wang et al., 2003; Moghimifam et al., 2020). For example, Moghimifam et al. (2020) found that elevated CO_2_ enhances CAT, polyphenol oxidase (PPO), SOD, and proline activity in algae (*Dunaliella* sp.). Our result may reflect that reduced CO_2_ often increases photorespiration (Moroney et al., 2013; Voss et al., 2013) and since photorespiration is the key source for ROS production (Voss et al., 2013), reduced CO_2_ may cause increased antioxidant enzyme activity in order to scavenge excessive ROS produced. However, these studies did not address whether Si played a role in these changes, while our findings suggest Si as an important influencer of the activity of these enzymes.

### Herbivory and Si enhanced antioxidant enzyme activity

In addition to CO_2_ having direct and Si-mediated impacts on antioxidant enzyme activity, it is also possible that the amount of herbivore damage played a role in antioxidant enzyme activity. Herbivores induced higher activity of CAT and APX, so these enzymes activity is at least associated with the levels of damage done to the plant, which has been similarly reported in previous studies (Leitner et al., 2005; Yang et al., 2017). Increased levels of antioxidant enzyme under reduced CO_2_ may reflect the fact that insects were doing less damage to the plant under these conditions and, therefore, there was less ROS oxidative stress that may have persisted in plant tissues to react with higher levels of antioxidant enzyme. Results from the ANCOVA and correlation tests also revealed that CO_2_ directly changed antioxidant enzyme activity and altered feeding efficiency (leaf consumption), which eventually reduced the growth rate of herbivores.

### Diminishing Si defence with rising CO_2_

The recent finding by Biru et al. (2021) showed that *H. armigera* RGR was lowest when fed on the model grass *Brachypodium distachyon* grown under reduced CO_2_ due to these plants having higher levels of Si defences compared to plants grown under ambient and elevated CO_2_ concentrations. In the current study, we observed that tall fescue had the highest concentrations of foliar N when grown under reduced CO_2_, which, in theory, could have promoted herbivore RGR because N is frequently the limiting factor in insect herbivore diets (Mattson, 1980; Huberty and Denno, 2006). The lower production of frass under reduced CO_2_, which we used as a proxy for plant consumption, suggests that herbivores were deterred from feeding; thus, they would have not been able to access these N resources.

Understanding the diminishing levels of Si-based plant defences against herbivores under future elevated atmospheric CO_2_ concentrations has received limited attention. Previous studies have shown that elevated CO_2_ concentrations decrease Si accumulation in different Poaceae genera (e.g., grass species and wheat) (Johnson and Hartley, 2018; Biru et al., 2022; Johnson et al., 2022), and this was associated with reduced Si defences while increasing herbivore performance; however, this effect is not always reproducible (Frew et al., 2017). The impact of elevated CO_2_ on Si defences reflects that plants switch from Si defences to C-based defences due to higher C availability under this scenario (Johnson and Hartley, 2018; Johnson et al., 2022). Although not examined in the context of Si defences, previous studies have demonstrated that elevated atmospheric CO_2_ increased consumption and growth rate of the generalist (*Pseudaletia unipuncta*) and specialist (*Spodoptera frugiperda*) insect herbivores when fed on C_3_ grass relative to lower atmospheric CO_2_ conditions (Barbehenn et al., 2004). Johnson et al. (2020) also reported that *H. armigera* RGR increased under elevated CO_2_ as a result of lower plant defence signalling and minimal reductions in the nutritional quality of lucerne (*Medicago sativa*).

## Conclusions

The present study provides further evidence that CO_2_ concentrations are strong drivers of Si accumulation in an important plant species, not previously reported on. We found strong evidence that reduced CO_2_ increased foliar Si concentration and antioxidant enzyme levels, which potentially linked to suppressed insect herbivore performance. This suggests that the negative effects of silicification, whether via physical or biochemical mechanisms, are stronger under reduced CO_2_. We showed a strong linkage between Si supplementation and activity of antioxidant enzyme, which may help in alleviating the harmful effects of herbivore-induced oxidative stress on plant defence responses. Although Si defences are minimal under elevated atmospheric CO_2_ conditions, many agricultural soils can become deficient in bioavailable Si (Haynes, 2017), which points to the importance of maintaining Si levels in soils under future projected atmospheric CO_2_ conditions.

## Data availability statement

The raw data supporting the conclusions of this article will be made available by the authors, without undue reservation.

## Author contributions

FB: Conceptualization, Data curation, Formal Analysis, Methodology, Validation, Writing – original draft. CC: Supervision, Writing – review & editing. RE: Supervision, Writing – review & editing. SJ: Conceptualization, Supervision, Writing – review & editing.

## References

[B1] Acevedo F. E. Peiffer M. Ray S. Tan C.-W. Felton G. W. (2021). Silicon-mediated enhancement of herbivore resistance in agricultural crops. Front. Plant Sci. 12, 631824. doi: 10.3389/fpls.2021.631824 PMC7928372 33679847

[B2] Ahanger M. A. Bhat J. A. Siddiqui M. H. Rinklebe J. Ahmad P. (2020). Integration of silicon and secondary metabolites in plants: a significant association in stress tolerance. J. Exp. Bot. 71, 6758–6774. doi: 10.1093/jxb/eraa291 32585681

[B3] Alhousari F. Greger M. (2018). Silicon and mechanisms of plant resistance to insect pests. Plants-Basel 7, 33. doi: 10.3390/plants7020033 29652790 PMC6027389

[B4] Aslam M. Albassam M. (2020). Presenting *post hoc* multiple comparison tests under neutrosophic statistics. J. King Saud Univ. Sci. 32, 2728–2732. doi: 10.1016/j.jksus.2020.06.008

[B5] Barbehenn R. V. Karowe D. N. Spickard A. (2004). Effects of elevated atmospheric CO_2_ on the nutritional ecology of C_3_ and C_4_ grass-feeding caterpillars. Oecologia 140, 86–95. doi: 10.1007/s00442-004-1572-9 15118901

[B6] Bi J. L. Felton G. W. (1995). Foliar oxidative stress and insect herbivory: primary compounds, secondary metabolites, and reactive oxygen species as components of induced resistance. J. Chem. Ecol. 21, 1511–1530. doi: 10.1007/bf02035149 24233680

[B7] Biru F. N. Cazzonelli C. I. Elbaum R. Johnson S. N. (2020). Contrasting effects of Miocene and Anthropocene levels of atmospheric CO_2_ on silicon accumulation in a model grass. Biol. Lett. 16, 20200608. doi: 10.1098/rsbl.2020.0608 33232651 PMC7728683

[B8] Biru F. N. Cazzonelli C. I. Elbaum R. Johnson S. N. (2022). Contrasting impacts of herbivore induction and elevated atmospheric CO_2_ on silicon defences and consequences for subsequent herbivores. Entomol. Exp. Appl. 170, 681–688. doi: 10.1111/eea.13168

[B9] Biru F. N. Islam T. Cibils-Stewart X. Cazzonelli C. I. Elbaum R. Johnson S. N. (2021). Anti-herbivore silicon defences in a model grass are greatest under Miocene levels of atmospheric CO_2_ . Glob. Change Biol. 27, 2959–2969. doi: 10.1111/gcb.15619 33772982

[B10] Cavalcanti F. R. Oliveira J. T. A. Martins-Miranda A. S. Viégas R. A. Silveira J. A. G. (2004). Superoxide dismutase, catalase and peroxidase activities do not confer protection against oxidative damage in salt-stressed cowpea leaves. New Phytol. 163, 563–571. doi: 10.1111/gcb.15619 33873746

[B11] Cooke J. Leishman M. R. (2011). Is plant ecology more siliceous than we realise? Trends Plant Sci. 16, 61–68. doi: 10.1016/j.tplants.2010.10.003 21087891

[B12] Coskun D. Deshmukh R. Sonah H. Menzies J. Reynolds O. Kronzucker. (2019). The controversies of silicon’s role in plant biology. New Phytol. 221, 1340–1357. doi: 10.1111/nph.15343 30007071

[B13] Craine J. M. (2009). Resource strategies of wild plants (Princeton, NJ, USA: Princeton University Press). doi: 10.1515/9781400830640

[B14] Das K. Roychoudhury A. (2014). Reactive oxygen species (ROS) and response of antioxidants as ROS-scavengers during environmental stress in plants. Front. Environ. Sci. 2, 53. doi: 10.3389/fenvs.2014.00053

[B15] Debona D. Rodrigues F. Datnoff L. (2017). Silicon’s role in abiotic and biotic plant stresses. Annu. Rev. Phytopathol. 55, 85–107. doi: 10.1146/annurev-phyto-080516-035312 28504920

[B16] Epstein E. (1994). The anomaly of silicon in plant biology. PNAS. 91, 11–17. doi: 10.1073/pnas.91.1.11 11607449 PMC42876

[B17] Epstein E. (2009). Silicon: its manifold roles in plants. Ann. Appl. Biol. 155, 155–160. doi: 10.1111/j.1744-7348.2009.00343.x

[B18] Farooq M. A. Ali S. Hameed A. Ishaque W. Mahmood K. Iqbal Z. (2013). Alleviation of cadmium toxicity by silicon is related to elevated photosynthesis, antioxidant enzymes; suppressed cadmium uptake and oxidative stress in cotton. Ecotoxicol. Environ. Saf. 96, 242–249. doi: 10.1016/j.ecoenv.2013.07.006 23911213

[B19] Fichman Y. Mittler R. (2020). Rapid systemic signaling during abiotic and biotic stresses: is the ROS wave master of all trades? TPJ. 102, 887–896. doi: 10.1111/tpj.14685 31943489

[B20] Fimognari L. Dölker R. Kaselyte G. Jensen C. N. G. Akhtar S. S. Großkinsky D. K.. (2020). Simple semi-high throughput determination of activity signatures of key antioxidant enzymes for physiological phenotyping. Plant Methods 16, 42. doi: 10.1186/s13007-020-00583-8 32206082 PMC7085164

[B21] Frew A. Allsopp P. G. Gherlenda A. N. Johnson S. N. (2017). Increased root herbivory under elevated atmospheric carbon dioxide concentrations is reversed by silicon-based plant defences. J. Appl. Ecol. 54, 1310–1319. doi: 10.1111/1365-2664.12822

[B22] Fulweiler R. W. Maguire T. J. Carey J. C. Finzi A. C. (2014). Does elevated CO_2_ alter silica uptake in trees? Front. Plant Sci. 5, 793. doi: 10.3389/fpls.2014.00793 PMC4292721 25628636

[B23] Gong H. J. Zhu X. Y. Chen K. M. Wang S. M. Zhang C. L. (2005). Silicon alleviates oxidative damage of wheat plants in pots under drought. Plant Sci. 169, 313–321. doi: 10.1016/j.plantsci.2005.02.023

[B24] Hall C. R. Dagg V. Waterman J. M. Johnson S. N. (2020). Silicon alters leaf surface morphology and suppresses insect herbivory in a model grass species. Plants 9, 643. doi: 10.3390/plants9050643 32438683 PMC7285219

[B25] Hall C. R. Waterman J. M. Vandegeer R. K. Hartley S. E. Johnson S. N. (2019). The role of silicon in antiherbivore phytohormonal signalling. Front. Plant Sci. 10, 1132. doi: 10.3389/fpls.2019.01132 PMC6759751 31620157

[B26] Han Y. Li P. Gong S. Yang L. Wen L. Hou M. (2016). Defense responses in rice induced by silicon amendment against infestation by the leaf folder *Cnaphalocrocis medinalis* . PloS One 11, e0153918. doi: 10.1371/journal.pone.0153918 27124300 PMC4849577

[B27] Hartley S. E. Fitt R. N. McLarnon E. L. Wade R. N. (2015). Defending the leaf surface: intra- and inter-specific differences in silicon deposition in grasses in response to damage and silicon supply. Front. Plant Sci. 6, 35. doi: 10.3389/fpls.2015.00035 PMC4324063 25717331

[B28] Hartmann J. Asch F. (2019). Extraction, storage duration, and storage temperature affect the activity of ascorbate peroxidase, glutathione reductase, and superoxide dismutase in rice tissue. Biology 8, 70. doi: 10.3390/biology8040070 31554149 PMC6956177

[B29] Hasanuzzaman M. Bhuyan M. H. M. B. Zulfiqar F. Raza A. Mohsin S. M. Mahmud J. A.. (2020). Reactive oxygen species and antioxidant defense in plants under abiotic stress: revisiting the crucial role of a universal defense regulator. Antioxidants 9, 681. doi: 10.3390/antiox9080681 32751256 PMC7465626

[B30] Hasanuzzaman M. Nahar K. Anee T. I. Khan M. I. R. Fujita M. (2018). Silicon-mediated regulation of antioxidant defense and glyoxalase systems confers drought stress tolerance in *Brassica napus L. S* . Afr. J. Bot. 115, 50–57. doi: 10.1016/j.sajb.2017.12.006

[B31] Haynes R. J. (2017). Chapter 3: Significance and role of Si in crop production. Adv. Agron. 146, 83–166. doi: 10.1016/bs.agron.2017.06.001

[B32] Hiltpold I. Demarta L. Johnson S. N. Moore B. D. Power S. A. Mitchell C.. (2017). “Silicon and other essential element composition in roots using X-ray fluorescence spectroscopy: A high throughput approach,” in Invertebrate ecology of Australasian grasslands. Ed. Johnson S. N. (Western Sydney University, Hawkesbury, NSW, Australia:Proceedings of the Ninth ACGIE. Western Sydney University), 191–196).

[B33] Hodson M. J. White P. J. Mead A. Broadley M. R. (2005). Phylogenetic variation in the silicon composition of plants. Ann. Bot. 96, 1027–1046. doi: 10.1093/aob/mci255 16176944 PMC4247092

[B34] Huang H. Ullah F. Zhou D.-X. Yi M. Zhao Y. (2019). Mechanisms of ROS regulation of plant development and stress responses. Front. Plant Sci. 10, 800. doi: 10.3389/fpls.2019.00800 PMC6603150 31293607

[B35] Huberty A. F. Denno R. F. (2006). Consequences of nitrogen and phosphorus limitation for the performance of two planthoppers with divergent life-history strategies. Oecologia 149, 444–455. doi: 10.1007/s00442-006-0462-8 16794833

[B36] IPCC (2014). Climate Change 2014: Impacts, Adaptation, and Vulnerability. Part A: Global and sectoral aspects. Contribution of working group II to the fifth assessment report of the intergovernmental panel on climate change. Eds. Field C. B. Baros V. R. Dokken D. J. Mach K. J. Mastrandrea M. D. Bilir T. E. Chatterjee M. Ebi K. L. Estrada Y. O. Genova R. C. Girma B. Kissel E. S. Levy A. N. MacCracken S. Mastrandrea P. R. White L. L. (Cambridge, UK and New York, NY: Cambridge University Press).

[B37] Islam T. Ben D M. S Cott N J. (2020). Novel evidence for systemic induction of silicon defences in cucumber following attack by a global insect herbivore. Ecol. Entomol. 45, 1373–1381. doi: 10.1111/een.12922

[B38] Johnson S. N. Barton C. V. M. Biru F. N. Islam T. Mace W. J. Rowe R. C.. (2023). Elevated atmospheric CO_2_ suppresses silicon accumulation and exacerbates endophyte reductions in plant phosphorus. Funct. Ecol. 37, 1567–1579. doi: 10.1111/1365-2435.14342

[B39] Johnson S. N. Cibils-Stewart X. Waterman J. M. Biru F. N. Rowe R. C. Hartley S. E. (2022). Elevated atmospheric CO_2_ changes defence allocation in wheat but herbivore resistance persists. Proc. R. Soc B: Biol. Sci. 289, 20212536. doi: 10.1098/rspb.2021.2536 PMC8848237 35168395

[B40] Johnson S. N. Hartley S. E. (2018). Elevated carbon dioxide and warming impact silicon and phenolic-based defences differently in native and exotic grasses. Glob. Change Biol. 102, 3886–3896. doi: 10.1002/ecy.3250 29105229

[B41] Johnson S. N. Ryalls J. M. W. Gherlenda A. N. Frew A. Hartley S. E. (2018). Benefits from below: silicon supplementation maintains legume productivity under predicted climate change scenarios. Front. Plant Sci. 9, 202. doi: 10.3389/fpls.2018.00202 PMC5829608 29527218

[B42] Johnson S. N. Waterman J. M. Hall C. R. (2020). Increased insect herbivore performance under elevated CO_2_ is associated with lower plant defence signalling and minimal declines in nutritional quality. Sci. Rep. 10, 14553. doi: 10.1038/s41598-020-70823-3 32883958 PMC7471906

[B43] Jung H.-I. Yan J. Zhai Z. Vatamaniuk O. (2015). Gene functional analysis using protoplast transient assays. In: Alonso, J., Stepanova, A. (eds) Plant Functional Genomics. Methods in Molecular Biology, vol 1284. Humana Press, New York, NY 10.1007/978-1-4939-2444-8_22 25757786

[B44] Kerchev P. I. Fenton B. Foyer C. H. Hancock R. D. (2012). Plant responses to insect herbivory: interactions between photosynthesis, reactive oxygen species and hormonal signalling pathways. Plant, Cell Environ. 35, 441–453. doi: 10.1111/j.1365-3040.2011.02399.x 21752032

[B45] Kim Y.-H. Khan A. Waqas M. Lee I.-J. (2017). Silicon regulates antioxidant activities of crop plants under abiotic-induced oxidative stress: a review. Front. Plant Sci. 8, 510. doi: 10.3389/fpls.2017.00510 PMC5382202 28428797

[B46] Kim Y. H. Khan A. L. Waqas M. Shahzad R. Lee I. J. (2016). Silicon-mediated mitigation of wounding stress acts by up-regulating the rice antioxidant system. Cereal Res. Commun. 44, 111–121. doi: 10.1556/0806.43.2015.031

[B47] Kumar S. Soukup M. Elbaum R. (2017). Silicification in grasses: variation between different cell types. Front. Plant Sci. 8, 438. doi: 10.3389/fpls.2017.00438 PMC5368260 28400787

[B48] Leitner M. Boland W. Mithöfer A. (2005). Direct and indirect defences induced by piercing-sucking and chewing herbivores in *Medicago truncatula* . New Phytol. 167, 597–606. doi: 10.1111/j.1469-8137.2005.01426.x 15998409

[B49] Ma J. F. Yamaji N. (2006). Silicon uptake and accumulation in higher plants. Trends Plant Sci. 11, 392–397. doi: 10.1016/j.tplants.2006.06.007 16839801

[B50] Maksimović J. J. D. Živanović B. D. (2012). Quantification of the antioxidant activity in salt-stressed tissues. Methods Mol. Biol. 913, 237–250. doi: 10.1007/978-1-61779-986-0_16 22895764

[B51] Massey F. P. Ennos A. R. Hartley S. E. (2006). Silica in grasses as a defence against insect herbivores: contrasting effects on folivores and a phloem feeder. J. Anim. Ecol. 75, 595–603. doi: 10.1111/j.1365-2656.2006.01082.x 16638012

[B52] Massey F. P. Hartley S. E. (2009). Physical defences wear you down: progressive and irreversible impacts of silica on insect herbivores. J. Anim. Ecol. 78, 281–291. doi: 10.1111/j.1365-2656.2008.01472.x 18771503

[B53] Massey F. P. Roland Ennos A. Hartley S. E. (2007). Herbivore specific induction of silica-based plant defences. Oecologia 152, 677–683. doi: 10.1007/s00442-007-0703-5 17375331

[B54] Mattson W. J. (1980). Herbivory in relation to plant nitrogen content. Annu. Rev. Evol. Syst. 11, 119–161. doi: 10.1146/annurev.es.11.110180.001003

[B55] Moghimifam R. Niknam V. Ebrahimzadeh H. Hejazi M. A. (2020). The influence of different CO_2_ concentrations on the biochemical and molecular response of two isolates of *Dunaliella* sp. (ABRIINW-CH2 and ABRIINW-SH33). J. Appl. Phycol. 32, 175–187. doi: 10.1007/s10811-019-01914-6

[B56] Moroney J. V. Jungnick N. DiMario R. J. Longstreth D. J. (2013). Photorespiration and carbon concentrating mechanisms: two adaptations to high O_2_, low CO_2_ conditions. Photosynth. Res. 117, 121–131. doi: 10.1007/s11120-013-9865-7 23771683

[B57] Moussa H. (2006). Influence of exogenous application of silicon on physiological response of salt-stressed maize (*Zea mays* L.). Int. J. Agric. Biol. 8, 293–297. doi: 10.1007/s12633-015-9372-x

[B58] Perry C. C. Mann S. Williams R. J. P. (1984). Structural and analytical studies of the silicified macrohairs from the lemma of the grass *Phalaris canariensis* L. Proc. R. Soc B. 222, 427–438. doi: 10.1098/rspb.1984.0075

[B59] Pizzino G. Irrera N. Cucinotta M. Pallio G. Mannino F. Arcoraci V.. (2017). Oxidative stress: harms and benefits for human health. Oxid. Med. Cell. Longev. 2017, 8416763. doi: 10.1155/2017/8416763 28819546 PMC5551541

[B60] Raven J. A. (1983). The transport and function of silicon in plants. Biol. Rev. 58, 179–207. doi: 10.1111/j.1469-185x.1983.tb00385.x

[B61] Reidinger S. Ramsey M. H. Hartley S. E. (2012). Rapid and accurate analyses of silicon and phosphorus in plants using a portable X-ray fluorescence spectrometer. New Phytol. 195, 699–706. doi: 10.1111/j.1469-8137.2012.04179.x 22671981

[B62] Reynolds O. L. Keeping M. G. Meyer J. H. (2009). Silicon-augmented resistance of plants to herbivorous insects: a review. Ann. Appl. Biol. 155, 171–186. doi: 10.1111/j.1744-7348.2009.00348.x

[B63] Reynolds O. L. Padula M. P. Zeng R. S. Gurr G. M. (2016). Silicon: potential to promote direct and indirect effects on plant defense against arthropod pests in agriculture. Front. Plant Sci. 7, 744. doi: 10.3389/fpls.2016.00744 PMC4904004 27379104

[B64] Ryalls J. M. W. Hartley S. E. Johnson S. N. (2017). Impacts of silicon-based grass defences across trophic levels under both current and future atmospheric CO_2_ scenarios. Biol. Lett. 13, 20160912. doi: 10.1098/rsbl.2016.0912 28298594 PMC5377035

[B65] Sharma P. Jha A. B. Dubey R. S. Pessarakli M. (2012). Reactive oxygen species, oxidative damage, and antioxidative defense mechanism in plants under stressful conditions. J. Bot. 2012, 1–26. doi: 10.1155/2012/217037

[B66] Sudhakar C. Lakshmi A. Giridarakumar S. (2001). Changes in the antioxidant enzyme efficacy in two high yielding genotypes of mulberry (*Morus alba* L.) under NaCl salinity. Plant Sci. 161, 613–619. doi: 10.1016/s0168-9452(01)00450-2

[B67] Teakle R. Jensen J. (1985). “Heliothis punctiger,” in Handbook of insect rearing. Eds. Singh P. Moore R. F. (Amsterdam, The Netherlands: Elsevier), 313–322.

[B68] Tripathy B. C. Oelmüller R. (2012). Reactive oxygen species generation and signaling in plants. Plant Signal. Behav. 7, 1621–1633. doi: 10.4161/psb.22455 23072988 PMC3578903

[B69] Voss I. Sunil B. Scheibe R. Raghavendra A. S. (2013). Emerging concept for the role of photorespiration as an important part of abiotic stress response. Plant Biol. 15, 713–722. doi: 10.1111/j.1438-8677.2012.00710.x 23452019

[B70] Wang S. Y. Bunce J. A. Maas J. L. (2003). Elevated carbon dioxide increases contents of antioxidant compounds in field-grown strawberries. J. Agric. Food Chem. 51, 4315–4320. doi: 10.1021/jf021172d 12848504

[B71] Waterman J. M. Hall C. R. Mikhael M. Cazzonelli C. I. Hartley S. E. Johnson S. N. (2020). Short-term resistance that persists: rapidly induced silicon anti-herbivore defence affects carbon-based plant defences. Funct. Ecol. 35, 82–92. doi: 10.1111/1365-2435.13702

[B72] Yang L. Han Y. Li P. Li F. Ali S. Hou M. (2017). Silicon amendment is involved in the induction of plant defense responses to a phloem feeder. Sci. Rep. 7, 4232. doi: 10.1038/s41598-017-04571-2 28652621 PMC5484686

